# Nanotechnology and Its Application in Dentistry: A Systematic Review of Recent Advances and Innovations

**DOI:** 10.3390/jcm13175268

**Published:** 2024-09-05

**Authors:** Gianna Dipalma, Alessio Danilo Inchingolo, Mariafrancesca Guglielmo, Roberta Morolla, Irene Palumbo, Lilla Riccaldo, Antonio Mancini, Andrea Palermo, Giuseppina Malcangi, Angelo Michele Inchingolo, Francesco Inchingolo

**Affiliations:** 1 Department of Interdisciplinary Medicine, University of Bari “Aldo Moro”, 70121 Bari, Italy; giannadipalma@tiscali.it (G.D.); ad.inchingolo@libero.it (A.D.I.); m.guglielmo2@studenti.uniba.it (M.G.); roberta.morolla@uniba.it (R.M.); irene.palumbo@uniba.it (I.P.); l.riccaldo@studenti.uniba.it (L.R.); dr.antonio.mancini@gmail.com (A.M.); giuseppinamalcangi@libero.it (G.M.); 2 College of Medicine and Dentistry, Birmingham B4 6BN, UK; andrea.palermo2004@libero.it

**Keywords:** nanotechnology, dentistry, nanomaterials, orthodontics, dental implants, enamel remineralization, antimicrobial properties, osseointegration, dental composites, restorative dentistry

## Abstract

**Background:** This study looks at the clinical applications of nanotechnology in dentistry, with an emphasis on implantology, preventive care, orthodontics, restorative dentistry, and endodontics. **Methods:** Following PRISMA criteria and registered in PROSPERO (ID: CRD 564245), a PubMed, Scopus, and Web of Science search was conducted for studies from January 2014 to April 2024. The criteria were English-language research on nanotechnology in dental coatings, with a focus on clinical trials and observational studies. The electronic database search yielded 8881 publications. Following the screening process, 17 records were selected for qualitative analysis. **Results:** Nanotechnology has revolutionized dentistry. In orthodontics, nanoparticles improve antibacterial characteristics, durability, and biocompatibility, lowering bacterial colonization and plaque. In preventative care, Casein Phosphopeptide-Amorphous Calcium Phosphate (CPP-ACP) combined with stannous fluoride (SnF_2_) and nano-sized sodium trimetaphosphate (TMPnano) substantially remineralizes enamel. Nanostructured surfaces in dental implants, particularly those containing calcium, improve osseointegration and stability. Nanoparticles in restorative dentistry improve composite and adhesive strength, aesthetics, and longevity. **Conclusions:** Nanotechnology improves dental materials and equipment, resulting in better treatment outcomes and increased patient comfort. Its integration provides more effective treatments, which improves dental care and patient outcomes. More research is needed to overcome present problems and expand nanotechnology’s medicinal applications.

## 1. Introduction

Nanotechnology, the manipulation of matter on an atomic, molecular, and supra-molecular scale, has revolutionized various scientific fields, including medicine and dentistry [1]. By enabling precise control over material properties and interactions at a nanoscopic level, nanotechnology has opened new avenues for innovation and improvement in these disciplines [2,3,4,5]. 

The integration of nanotechnology in dentistry, involving the use of nanomaterials and nanoparticles, has paved the way for significant advancements in dental care and treatment [6,7,8,9,10]. This burgeoning field, known as nanodentistry, holds the potential to transform conventional dental practices, offering novel solutions for diagnostics, therapeutics, and regenerative procedures [11,12,13,14].

Nanomaterials are materials with one or more external dimensions in the size range 1 nm–100 nm. Their unique properties, such as increased surface area-to-volume ratio, quantum effects, and enhanced mechanical, electrical, and optical characteristics, distinguish them from their bulk counterparts [15,16,17,18]. Nanoparticles, defined as having one or more dimensions of 1–100 nm, are employed in various dental applications due to their bioactive properties, antimicrobial activity, and ability to interact at the molecular level [19,20]. These characteristics make them particularly suited for addressing complex dental issues, from targeted drug delivery systems to improved dental implants and restorative materials [21,22,23,24,25,26,27].

### 1.1. Application of Nanotechnology in Dentistry

The concept of nanotechnology was first introduced by physicist Richard Feynman in 1959, but it was not until the advent of sophisticated imaging and manipulation techniques, such as atomic force microscopy (AFM) and scanning tunneling microscopy (STM), that the field truly began to flourish [28,29].

These technological advancements allowed scientists to observe and manipulate matter at the nanoscale with unprecedented precision. In dentistry, the application of nanotechnology started gaining momentum in the early 2000s, leading to the development of nanocomposites, nano-coatings, and nanoparticles for various dental treatments. For instance, nanocomposites have shown improved mechanical properties and aesthetics, while nano-coatings can enhance the surface properties of dental implants, making them more resistant to bacterial colonization and improving osseointegration [2,12,30,31,32].

### 1.2. Challenges and Future Directions

Despite the promising advancements, the integration of nanotechnology in dentistry faces several challenges. The long-term biocompatibility and safety of nanomaterials, potential cytotoxicity, and environmental impact require thorough investigation. The small size and high reactivity of nanoparticles can lead to unintended interactions with biological systems, which may cause damage to oral tissues or cells, leading to inflammation or allergic reactions [33,34]. These concerns are critical, as the interaction between nanomaterials and biological systems can have unforeseen effects, necessitating comprehensive studies to ensure patient safety [35,36,37]. Additionally, the long-term effects of nanoparticle accumulation in the body remain largely unknown, posing potential risks to overall health [38].

Regulatory approvals and standardization of nanotechnology-based dental products are essential to ensure their safe and effective use. This involves developing clear guidelines and protocols for the production, testing, and clinical application of nanodental products. Moreover, the high cost of nanomaterials and the need for specialized equipment and training may limit their widespread adoption in clinical practice [39]. 

Future research should focus on developing multifunctional nanomaterials with enhanced properties, exploring new applications of nanotechnology in dental diagnostics and therapeutics, and addressing the existing challenges to pave the way for the broader implementation of nanodentistry. This includes exploring the potential of nanotechnology in areas such as implantology, where nanomaterials can improve the integration and longevity of dental implants, and preventive care, where nanoscale coatings can protect teeth from decay and erosion [36,40,41]. 

Nanotechnology holds immense potential to revolutionize the field of dentistry, offering innovative solutions for implantology, preventive care, orthodontics, restorative dentistry, and endodontics. The unique properties of nanomaterials and nanoparticles enable the development of advanced dental products and techniques that enhance treatment efficacy, patient comfort, and clinical outcomes. 

As research in nanodentistry progresses, addressing the associated challenges and ensuring the safe and effective integration of nanotechnology into clinical practice will be paramount. 

The objective of this systematic review is to comprehensively explore and elucidate the diverse applications and implications of nanotechnology in various dental specialties. By conducting an in-depth analysis of the integration of nanomaterials and nanoparticles in orthodontics, preventive and community dentistry, dental implants, restorative dentistry, and endodontics, this review seeks to provide a detailed understanding of how nanotechnology can revolutionize dental care and treatment. It aims to explore and elaborate on the clinical aspects and implications of nanotechnology across these specialties, such as enhancing the integration and durability of dental implants in implantology, advancing protective coatings and treatments to prevent decay and disease in preventive dentistry, developing more effective and less invasive treatment options in orthodontics, improving the properties and performance of restorative materials in restorative dentistry, and offering innovative solutions for root canal treatments and tissue regeneration in endodontics. The review highlights the unique properties of nanomaterials that contribute to improved treatment outcomes, patient comfort, and long-term success of dental procedures, while also addressing the challenges and potential of nanotechnology in overcoming conventional limitations by enhancing biocompatibility, antimicrobial efficacy, and mechanical properties of dental materials. Ultimately, this review aspires to offer valuable insights for clinicians, researchers, and policymakers in the pursuit of advanced and effective dental care solutions.

## 2. Materials and Methods

### 2.1. Protocol and Registration 

This systematic review was conducted according to Preferred Reporting Items for Systematic Reviews and Meta-Analyses (PRISMA) and the protocol was registered at PROSPERO under the ID: CRD42024581880 [42]. 

### 2.2. Search Processing 

A search on PubMed, Scopus, and Web of Science was performed to find papers dating from 1 January 2014 to 1 April 2024. The search strategy used the Boolean keywords: (“nanotechnology” OR “nanoparticles” OR “nanostructures” OR “nanomaterials”) AND (“dental” OR “dentistry” OR “dental materials” OR “dental surfaces”) AND (“coating” OR “surface”) (Table 1).

### 2.3. Inclusion Criteria

The following inclusion criteria were considered: (1) studies that investigated the use of nanotechnology and nanomaterials as coatings in different fields of dentistry; (2) randomized clinical trials, retrospective studies, case-control studies and prospective studies; (3) in vitro studies; (4) English language, and (5) full-text.

Papers that did not match the above criteria were excluded.

The review was conducted using the PICO criteria:

- Participants: adults, both male and female;
- Interventions: use of nanotechnology and nanomaterials as coatings in various fields of dentistry. Nanomaterials are emerging as a promising solution in the field of dental regeneration due to their unique properties, such as small size, high specific surface area, and ability to interact with biological cells and tissues;
- Comparisons: different dentistry field;
- Outcomes: nanotechnology enhances dental materials’ properties, improving treatments and patient outcomes.

The PICO’s question of this systematic review is to comprehensively explore and elucidate the diverse applications and implications of nanotechnology in various dental specialties. 

### 2.4. Exclusion Criteria

The exclusion criteria were as follow: (1) animal studies; (2) off-topic; (3) reviews, case reports, case series, letters, or comments; (4) no English language. 

### 2.5. Data Processing 

Three reviewers (M.G., I.P. and R.M.) independently consulted the databases to collect the studies and rated their quality, based on selection criteria. The selected articles were downloaded into Zotero (version 6.0.15). Any divergence between the three authors was settled by discussion with a senior reviewer (F.I.).

### 2.6. Quality Assessment

The quality of the included papers was assessed by two reviewers, A.P. and A.M. using the ROBINS tool. Seven points were evaluated and each was assigned a degree of bias. A third reviewer (F.I.) was consulted in the event of a disagreement until an agreement was reached. The question in the domains evaluated in the ROBINS is the following:

- Bias due to confounding
- Bias arising from measurement of exposure
- Bias in the selection of participants into the study
- Bias due to post-exposure intervention
- Bias due to missing data
- Bias arising from measurement of the outcome
- Bias in the selection of the reported results

## 3. Results

### 3.1. Study Selection and Characteristics 

A total of 8881 publications were found using the electronic database search (Scopus N = 3203, PubMed N = 2977, Web of Science N = 2701); no articles were found using the manual search. Following the removal of duplicates (N = 1457), the titles and abstracts of 7424 studies were assessed for screening. A total of 298 records were chosen out of 7126 papers that did not match the inclusion requirements (6881 off-topic, 214 reviews, and 31 animal research). Following the exclusion of six records that could not be obtained, 277 reports were eliminated for failing to meet the inclusion requirements (263 off-topic, 14 reviews). In total, 15 records were chosen for qualitative investigation after being deemed eligible. 

**Figure 1:**
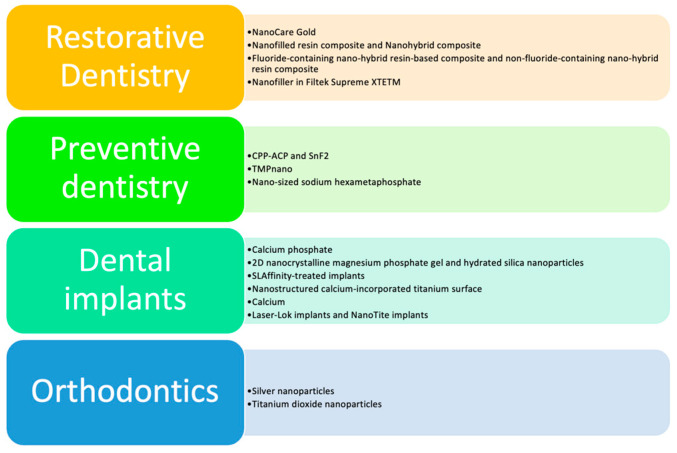
Applications of Nanotechnologies and Biomaterials in Dentistry.

The findings from the included studies highlight the effectiveness of nanotechnologies across various dental applications. In orthodontic patients, the incorporation of nanoparticles, such as silver and titanium dioxide, significantly reduced bacterial colonies, thereby enhancing oral hygiene. In the context of enamel remineralization, the use of agents like TMPnano and HMPnano resulted in increased enamel surface hardness and fluoride uptake. For dental implants, nanostructured surfaces promoted better osseointegration and long-term stability. Additionally, in restorative dentistry, nanocomposites improved mechanical strength and reduced the risk of secondary caries, contributing to more durable and high-quality dental restorations (Figure 1). 

Orthodontics: Farhadian et al. (2016) demonstrated that silver nanoparticles in orthodontic retainers significantly reduced Streptococcus mutans colonies after 7 weeks [43]. Similarly, Elabd et al. (2024) reported a significant reduction in bacterial load using titanium dioxide nanoparticles in orthodontic functional appliances [44].

Enamel Remineralization: Fernando et al. (2019) and Danelon et al. (2015, 2018) showed that the use of nanoparticles like CPP-ACP, SnF_2_, TMPnano, and HMPnano increased enamel hardness and remineralization capacity compared to conventional fluoride toothpaste [45,46,47].

Dental Implants: Mangano et al. (2017) reported improved early bone healing with calcium phosphate nanostructured implants [48]. Al-Hashedi et al. (2019) demonstrated the effectiveness of hydrated silica nanoparticles in decontaminating dental implants, while Keng-Liang Ou et al. (2016) observed enhanced osseointegration with SLAffinity-treated implants [49,50]. Felice et al. (2015) and Bechara et al. (2017) highlighted good long-term clinical outcomes with nanostructured implant surfaces [51,52].

Restorative Dentistry: Gomes Torres et al. (2014) and Nemt-Allah et al. (2021) demonstrated the effectiveness of silicon dioxide nanoparticles and NanoCare Gold in direct restorations [53,54]. Burke et al. (2017) reported 100% retention and no fractures with Filtek Supreme XTETM [55].

Endodontics: Elbahary et al. (2020) observed increased surface roughness in teeth treated with nanoparticles compared to controls [56].

**Figure 2:**
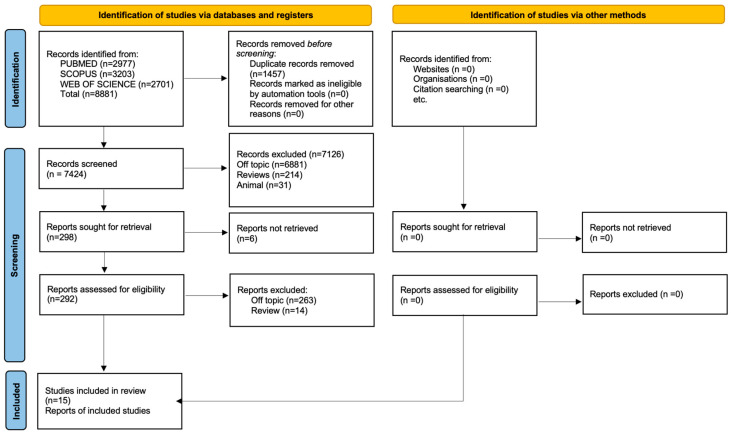
Literature search Preferred Reporting Items for Systematic Reviews and Meta-Analyses (PRISMA) flow diagram and database search indicators.

Figure 2 and Table 2 present the selection procedure and the summary of the selected records, respectively.

### 3.2. Quality Assessment and Risk of Bias of Included Articles

**Figure 3:**
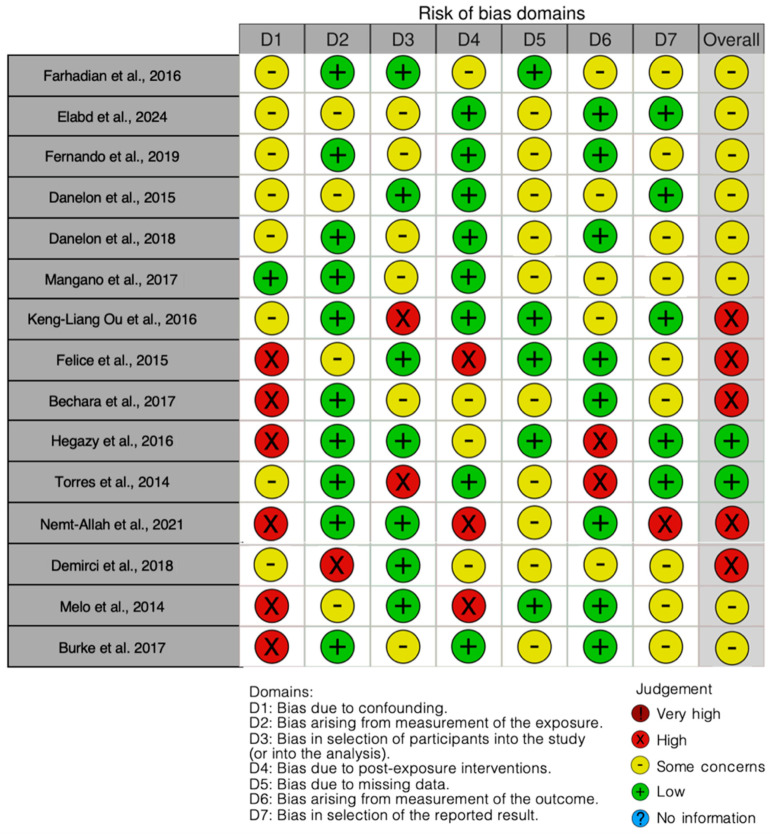
Bias assessment [46,47,48,49,50,51,52,53,54,55,56,57,58,59,60].

The risk of bias in the included studies is reported in Figure 3. Regarding the bias due to confounding, most studies exhibit some concerns. The bias arising from measurement of the exposure generally has a low risk of bias. Many studies also display a low risk of bias in the selection of participants. The bias due to missing data presents mostly some concerns. The bias arising from the measurement of the outcome is primarily low. The bias in the selection of the reported results mainly raises some concerns. The final results indicate that out of 17 analyzed articles, 4 studies have a low risk of bias, 8 studies have some concerns, and 5 studies have a high risk of bias.

## 4. Discussion

### 4.1. Use of Biomaterials and Nanotechnologies in Orthodontics

Nanotechnology has brought significant advancements to orthodontics, particularly by enhancing the properties of orthodontic devices [60]. The incorporation of nanomaterials, such as nanosilver particles, into adhesives, brackets, and retainers has not only improved their antimicrobial properties but also increased their durability and biocompatibility. These enhancements are crucial in addressing common orthodontic challenges, such as bacterial colonization and plaque accumulation, which can adversely affect treatment outcomes and patient comfort [60,61,62].

Beyond their antimicrobial properties, nanosilver particles also play a role in enhancing the structural integrity of orthodontic materials. The increased durability reduces the frequency of appliance failure and maintenance, which is a critical aspect for patient adherence and overall satisfaction. This multifaceted benefit underscores the importance of continued research into the optimization of nanoparticle size, shape, and concentration to maximize clinical efficacy while ensuring patient safety [61,62].

For example, conventional Hawley removable appliances, although popular for their esthetics and ease of use, are prone to issues like plaque accumulation and enamel demineralization due to their rough surfaces [43]. The study by Farhadian et al. demonstrated that adding nanosilver particles to orthodontic retainers effectively reduces Streptococcus mutans counts, thereby improving oral hygiene during retention periods. This finding underscores the potential of nanosilver particles in mitigating the adverse effects associated with traditional orthodontic appliances.

It is important to note, however, that the success of such interventions depends on careful consideration of the balance between antimicrobial efficacy and biocompatibility. As Farhadian et al. suggested, the effectiveness of nanosilver is contingent upon its size and concentration, which must be optimized to avoid potential cytotoxicity. Future studies should aim to establish standardized guidelines for the safe and effective use of these materials in clinical settings [43].

Similarly, Elabd et al.’s research on the use of titanium dioxide nanoparticles in twin block appliances further supports the role of nanotechnology in orthodontics. Their findings revealed a significant reduction in bacterial colonies when 1% titanium dioxide nanoparticles were incorporated into the appliances, suggesting an improvement in oral hygiene over time. These studies collectively highlight the promising impact of nanotechnology in enhancing the effectiveness and efficiency of orthodontic treatments [44].

While the antibacterial properties of titanium dioxide nanoparticles are promising, Elabd et al.’s study also points to the need for long-term clinical trials to assess the sustainability of these benefits. The potential for bacterial resistance to develop over extended use of nanoparticle-enhanced materials is a concern that warrants further investigation. Additionally, understanding the patient’s response to these materials over time will be crucial in determining their long-term viability in orthodontic practice [44].

As nanotechnology continues to evolve, its application in orthodontics is likely to expand, offering new solutions to existing challenges. Future research should focus on optimizing the use of nanomaterials in orthodontics, ensuring their safety, and exploring their potential in improving long-term treatment outcomes.

### 4.2. Use of Biomaterials and Nanotechnologies in Preventive and Community Dentistry

The escalating prevalence of dental caries, erosion, and hypersensitivity has catalyzed the pursuit of advanced preventive and treatment modalities in dentistry. This comprehensive review delves into the realm of nanotechnology for enamel remineralization, highlighting the potential of innovative approaches in addressing these pervasive oral health challenges [63,64].

Fernando et al. conducted a study that explored the integration of nanotechnology in dentistry, with a particular focus on the combined application of Casein Phosphopeptide-Amorphous Calcium Phosphate (CPP-ACP) and Stannous Fluoride (SnF_2_) for enamel remineralization [45]. Dental caries, erosion, and hypersensitivity are significant global concerns, driven by bacterial acid production and dietary factors, which make effective remineralization strategies imperative. While traditional agents like fluoride have been instrumental in aiding remineralization, they present limitations in the delivery of bioavailable ions. Novel approaches such as CPP-ACP have shown promise in stabilizing these ions and enhancing remineralization [45].

In another significant study, Danelon et al. in 2015 investigated the remineralizing potential of fluoride toothpaste supplemented with nano-sized sodium trimetaphosphate (TMPnano). This study suggest that the inclusion of nano-sized TMP in fluoride toothpaste significantly enhances its remineralization potential [46]. 

Building on this, Danelon et al. in 2019 assessed the remineralizing efficacy of a conventional toothpaste (1100 ppm F) fortified with nano-sized sodium hexametaphosphate (HMP-nano) on artificial caries lesions in situ [47]. This double-blinded crossover study, found that brushing with 1100 F/HMPnano significantly increased enamel surface hardness, subsurface hardness, and mineral recovery compared to 1100 F alone [47]. 

The enhanced remineralization observed with HMPnano suggests that such nano-enhanced formulations could be particularly beneficial in patients at high risk of dental caries. Nevertheless, the study highlights the need for further research to explore the long-term benefits and any potential side effects of prolonged use of these nano-enhanced toothpaste formulations. Additionally, understanding patient perceptions and acceptance of these new products will be crucial for their successful integration into routine dental care.

### 4.3. Use of Biomaterials and Nanotechnologies in Dental Implants

Research on the use of biomaterials and nanotechnologies in dental implants is continuously evolving, with numerous studies exploring how nanostructured surfaces and other advanced techniques can enhance osseointegration and the long-term success of implants. Below, six significant studies in this field are compared, analyzing the results and clinical implications of the different methodologies employed [65].

Mangano et al. analyzed the early bone response to immediately loaded temporary transmucosal implants in the posterior maxilla. Implants with a nanostructured calcium-incorporated (NCI) surface showed significantly higher bone-to-implant contact (BIC) compared to machined (MA) surface implants. This finding suggests that nanostructured surfaces can improve early bone healing under immediate loading conditions, a result supported by other studies highlighting the importance of nanotechnologies in promoting osseointegration [48]. The enhanced BIC observed with NCI surfaces may be attributed to the increased surface area and the bioactive properties of calcium, which promote osteoblast activity and faster bone regeneration [48]. 

Similarly, Bechara et al. reported a 99.1% survival rate for implants with nanostructured surfaces incorporating calcium ions in fully edentulous patients. These implants demonstrated enhanced osseointegration with minimal peri-implant bone resorption, suggesting that nanostructured surfaces not only promote early bone healing but also contribute to the long-term stability of implants [52].

The importance of nanotechnologies extends beyond promoting osseointegration. Keng et al. conducted a comprehensive evaluation of the effects of StemBios (SB) cell therapy and SLAffinity-treated implants on osseointegration. SLAffinity-treated implants, characterized by a nanostructured surface, showed improved stress distribution and bone cell adhesion. The combination of SB cell therapy and SLAffinity surface treatment further enhanced bone healing and implant stability, suggesting an integrated approach that leverages both nanotechnologies and advanced cell therapies [50].

Felice et al. compared titanium implants with nanostructured calcium-incorporated surfaces to implants with resorbable blast media (RBM) surfaces. The three-year clinical results showed no significant differences between the two types of implants, suggesting that both surface treatments are effective in the long term. However, the preference for one surface over the other might depend on specific clinical factors and patient needs [51].

Similarly, Hegazy (2016) compared peri-implant outcomes of laser-collar-treated implants and nanostructured surface implants. No significant differences were observed between the two implant types, indicating that both surface treatments provide comparable clinical performance in early loading protocols [57].

The analyzed studies highlight the crucial role of nanotechnologies in enhancing osseointegration and maintaining implant surface health. Nanostructured surfaces, especially those incorporating calcium, demonstrate superior capacity to promote early bone healing and long-term implant stability. Additionally, integrating advanced cell therapies and specific surface treatments can further improve clinical outcomes.

While some studies show no significant differences between different surface treatments, the continued exploration of nanotechnologies remains crucial for advancing the field of dental implants. As the body of evidence grows, it is likely that specific nanostructured surfaces or combinations of treatments will emerge as the gold standard for certain clinical situations. Ongoing research should focus on refining these technologies, reducing costs, and improving accessibility to ensure that the benefits of nanotechnology in dental implants can be realized by a broader patient population.

### 4.4. Use of Biomaterials and Nanotechnologies in Restorative Dentistry

The advent of nanotechnology has significantly advanced the field of restorative dentistry. Nanoparticles, due to their exceptional physical, chemical, and biological properties, have been incorporated into various dental materials to enhance their performance. In restorative dentistry, nanoparticles are used to improve the mechanical strength, aesthetic quality, and durability of dental composites and adhesives [66]. They also contribute to better wear resistance and polish retention, crucial for the longevity of dental restorations. Additionally, the antibacterial properties of certain nanoparticles help in reducing the risk of secondary caries, further promoting oral health [67].

A study conducted by Gomes Torres evaluated the effectiveness of two restorative materials: GrandioSO (a conventional composite) and GrandioSO Heavy Flow (a flowable composite) with different viscosity. Both materials demonstrated acceptable clinical performance over the 24-month period, showing no significant differences in color match, marginal discoloration, wear, marginal adaptation, proximal contact, postoperative sensitivity, and retention. Gomes Torres’ study suggest that both conventional and flowable composites can achieve satisfactory clinical outcomes over time, providing flexibility in material choice based on specific clinical situations [53]. 

In another clinical trial, three parallel groups were studied, with patients randomly allocated to receive NanoCare Gold, chlorhexidine, or no surface pretreatment [54]. NanoCare Gold exhibited promising results for the durability of direct tooth-colored restorations. The study on NanoCare Gold highlights the potential of nanoparticle-based pretreatment solutions to enhance the longevity and quality of restorations. The reduced marginal staining and improved marginal adaptation observed with NanoCare Gold suggest that incorporating nanoparticles into pretreatment protocols could offer a valuable strategy for improving the durability of restorations, particularly in esthetically demanding areas. However, larger-scale studies and long-term follow-up are necessary to confirm these findings and determine the best application practices [54].

Additionally, a study assessed the use of nanotechnology in class IV restorations, using Filtek Supreme XT (nanofilled) and Ceram X duo (nanohybrid) composites over five years [58]. The study showed no significant differences in clinical performance, with restoration fracture being the primary cause of failure. This suggests that modern nanocomposites offer promising long-term outcomes in dental restorations [58].

The five-year follow-up study on class IV restorations underscores the durability of modern nanocomposites in high-stress areas. The comparable performance of nanofilled and nanohybrid composites suggests that both types of materials can be effectively used in restorations subject to significant mechanical loads. The primary cause of failure being restoration fracture indicates that future research could focus on improving the fracture resistance of these materials, potentially through further refinement of their nanoparticle content or the development of novel composite formulations [58].

Furthermore, a single-center prospective study evaluated the anti-caries efficacy and surface roughness of fluoride-releasing (F-CR) and non-fluoride (CR) nano-hybrid resin-based composites. F-CR significantly increased fluoride concentrations and reduced demineralization compared to CR, without affecting surface roughness [59]. This study is particularly significant for preventive dentistry, as it demonstrates the dual benefit of these materials in enhancing both restorative and preventive outcomes. The ability of F-CR to increase fluoride concentrations while maintaining surface smoothness suggests that these materials could play a key role in reducing secondary caries risk, especially in high-caries-risk patients. Further studies could explore the long-term caries prevention benefits of these materials in different patient populations and clinical settings [59].

Finally, a study by the PREP Panel assessed the clinical performance of restorations using Filtek Supreme XT and Adper Easy Bond. Over three years, 75 restorations were evaluated by experienced clinicians, showing optimal outcomes with no secondary caries and optimal surface quality. Selective enamel etching was found to reduce marginal discoloration while maintaining marginal integrity, supporting the durability and aesthetic performance of the materials in clinical settings [55]. The use of selective enamel etching with Filtek Supreme XT highlights a technique that can enhance marginal integrity, reducing the risk of discoloration over time. These results suggest that combining advanced nanocomposite materials with optimized bonding protocols could further improve restorative outcomes, particularly in cases where aesthetics are paramount [55,68].

### 4.5. Limitations

The included studies varied widely in their designs, ranging from randomized controlled trials to observational and crossover studies. This heterogeneity complicates the comparison and synthesis of outcomes. Furthermore, the studies assessed diverse endpoints, such as bacterial colony counts, enamel remineralization, and implant osseointegration, which are not directly comparable.

Many studies featured small sample sizes, such as the eight participants in Fernando et al. (2019) [45] and the twelve participants in the studies by Danelon et al. (2015, 2018) [46,47]. Additionally, several studies did not provide detailed demographic information, which limits the generalizability of the findings across different populations.

Some studies did not provide explicit details about the methodologies used, such as the average age and gender of participants (e.g., Farhadian et al., 2016 [43]; Elabd et al., 2024 [44]). This lack of information hinders the ability to fully evaluate the study’s context and potential biases. 

The properties of nanoparticles, including size, concentration, and type, varied significantly among the studies. For instance, the studies investigated nanoparticles such as silver, titanium dioxide, calcium phosphate, and nanocomposites, each with distinct characteristics and mechanisms of action. This variability may influence the outcomes and limits the ability to draw consistent conclusions about the efficacy of nanotechnology in dental applications. 

Many studies had relatively short follow-up periods, ranging from a few weeks to several months. Long-term outcomes are essential for evaluating the sustained effectiveness and safety of nanomaterials in dentistry. The limited follow-up data may not adequately capture potential long-term benefits or adverse effects.

## 5. Conclusions

Nanotechnology has profoundly impacted dentistry by improving the properties of various dental materials and devices, offering significant advancements across multiple areas such as orthodontics, preventive care, dental implantology, and restorative dentistry. These innovations promise more effective and efficient treatments, paving the way for improved patient outcomes. However, it is crucial to acknowledge the potential for adverse reactions associated with the use of nanomaterials.

## Figures and Tables

**Table 1:** Database search indicators.

Database	Search Field	Results (Number of Papers Found)
PubMed	(“nanotechnology” OR “nanoparticles” OR “nanostructures” OR “nanomaterials”) AND (“dental” OR “dentistry” OR “dental materials” OR “dental surfaces”) AND (“coating” OR “surface”).	2977
Scopus	(“nanotechnology” OR “nanoparticles” OR “nanostructures” OR “nanomaterials”) AND (“dental” OR “dentistry” OR “dental materials” OR “dental surfaces”) AND (“coating” OR “surface”).	3203
Web of Science	(“nanotechnology” OR “nanoparticles” OR “nanostructures” OR “nanomaterials”) AND (“dental” OR “dentistry” OR “dental materials” OR “dental surfaces”) AND (“coating” OR “surface”).	2701

**Table 2:** Descriptive summary of item selection.

Author (Year)	Study Design	Number of Patients	Average Age and Gender	Observation Time	Application Field	Nanoparticle Involved	Outcomes
Farhadian et al., 2016 [43]	Randomized controlled trial	66 orthodontic patients	Not provided	7 weeks	Orthodontic retainers	Silver nanoparticles (approximately 40 nm in size and 500 ppm in concentration)	Silver nanoparticles showed a significant reduction in Streptococcus mutans colony-forming units after 7 weeks, demonstrating strong antimicrobial effects under clinical conditions.
Elabd et al., 2024 [44]	Randomized controlled trial	26 orthodontic patients	Not provided	6 months	Orthodontic functional appliances (twin block appliances)	Titanium dioxide nanoparticles (1% concentration in acrylic baseplates)	The test group showed a significant decrease in bacterial colony count compared to the control group, with a higher reduction in the six-month group compared to the baseline group.
Fernando et al., 2019 [45]	Randomized controlled trial	Eight healthy adults patients	The average age of the participants was 43 years, with an equal distribution of four M and four F.	10 days	Enamel remineralization as a preventive measure	The nanoparticles investigated were CPP-ACP and SnF_2_.	The effectiveness of CPP-ACP and SnF_2_ in enhancing enamel remineralization, as well as the synergistic effects observed when combining these agents.
Danelon et al., 2015 [46]	Double-blinded crossover study.	12 patients	Not provided	4 phases of 3 day each	Enamel remineralization as a preventive measure	TMPnano	Increased enamel surface hardness, higher remineralizing capacity, and increased enamel fluoride uptake with TMPnano-enhanced toothpaste compared to conventional fluoride toothpaste.
Danelon et al., 2018 [47]	Double-blinded crossover study.	12 patients	Not provided	4 phases of 3 day each	Enamel remineralization as a preventive measure	HMPnano	Significant increases in enamel surface hardness, subsurface hardness, and mineral recovery were observed with 1100 F/HMPnano compared to 1100 F alone. Enamel fluoride uptake was similar across groups, except placebo.
Mangano er al., 2017 [48]	Randomized controlled trial	15 patients	Mean age 57.9 ± 6.7 years (6 M; 9 F)	8 weeks	Dental implants	Calcium phosphate	Significantly higher BIC in NCI implants compared to other implants, suggesting enhanced early bone healing with nanostructured surfaces under immediate loading conditions.
Keng-Liang Ou et al., 2016 [50]	Clinical trial	11 patients	Average age 41.74 ± 9.14 years; Gender not specified	3 months	Dental implants	SLAffinity-treated implants	Enhanced bone healing and osseointegration, 100% success rate of implants, significantly higher bone densities with SB cell therapy compared to without therapy
Felice et al., 2015 [51]	Randomized controlled trial	60 patients	Not provided	12 weeks	Dental implants	Nanostructured calcium-incorporated titanium surface (Xpeed; MegaGen)	Both implant surfaces provided good clinical results with no significant difference in implant failure, prosthesis failure, complications, or peri-implant marginal bone loss over a three-year period.
Bechara et al., 2017 [52]	Retrospective study	56 patients	Average age of 61.4 years, predominantly M (57.1%)	3 years	Dental implants	Calcium	High short-term success with implant survival rates of 95.9% patient-based and 99.1% implant-based, minimal peri-implant marginal bone loss (0.49 ± 0.52 mm mesial and 0.53 ± 0.52 mm distal)
Hegazy et al., 2016 [57]	Prospective clinical trial	36 patients	The patients ranged in age from 47 to 78 years, with 24 men and 12 women selected	2 weeks	Dental implants	Laser-Lok implants (Laser collar–treated implants) and NanoTite implants (Nanosurface–treated implants).	Peri-implant outcomes, including MPI, MBI, PD, implant mobility, MBL, and peri-implant tissue stability.
Gomes Torres et al., (2014) [53]	Clinical trial	47 patients	Not provided	2 years	Restorative Dentistry	Nanoparticles of Silicon dioxide (SiO_2_) (20–40 nm)	Both materials showed acceptable clinical performance over 24 months.
Nemt-Allah et al., (2021) [54]	Randomized controlled trial	57 patients	17–50 y	2 years	Restorative Dentistry	NanoCare Gold	NanoCare Gold showed promising results for direct tooth-colored restorations
Demirci et al., (2018) [58]	Observational study	34 patients	27.1 y 9 M And 25 F	5 years	Restorative Dentistry	Nanofilled resin composite (Filtek Supreme XT), Nanohybrid composite (Ceram X duo)	No statistically significant differences between the composites
Melo et al., (2014) [59]	Prospective split-mouth study	20 patients	Mean age 26.4 years, 14 F and 6 M	2 years	Restorative Dentistry	F-CR and non-fluoride-containing nano-hybrid resin composite	F-CR reduced enamel demineralization There was no significant difference in surface roughness between the materials.
Burke et al., 2017 [55]	Randomized clinical trial	35 patients	53.9 y mean age. 27 F and 8 M	3 years	Restorative Dentistry	Nanofiller in Filtek Supreme XTETM	100% retention and lack of fracture; excellent surface quality and minimal surface roughness

BIC: bone-to-implant contact; CPP-ACP: Casein Phosphopeptide-Amorphous Calcium Phosphate; HMPnano: nano-sized sodium hexametaphosphate; MBI: modified bleeding index; MBL: marginal bone loss; MPI: modified plaque index; NCI: nanostructured calcium-incorporated; PD: peri-implant probing depth; TMPnano: Nano-sized sodium trimetaphosphate; SiO_2_: Silicon dioxide; SnF_2_: Stannous Fluoride.

## Data Availability

No new data were created or analyzed in this study.

## Abbreviations

BIC	bone-to-implant contact
CPP-ACP	Casein Phosphopeptide-Amorphous Calcium Phosphate
CR	non-fluoride composite
F	female
F-CR	fluoride-releasing composite
HMP	sodium hexametaphosphate
HMPnano	nano-sized sodium hexametaphosphate
M	male
MA	machined surface
NCI	nanostructured calcium-incorporated
NMP	nanocrystalline magnesium phosphate
RBM	resorbable blast media
SB	StemBios
SnF_2_	Stannous Fluoride
TMP	Trimetaphosphate
TMPnano	Nano-sized sodium trimetaphosphate
USPHS	US Public Health Service
